# A Self-Driven Microfluidic Chip for Ricin and Abrin Detection

**DOI:** 10.3390/s22093461

**Published:** 2022-05-02

**Authors:** Xuexin Bai, Chenyi Hu, Liang Chen, Jing Wang, Yanwei Li, Wei Wan, Zhiying Jin, Yue Li, Wenwen Xin, Lin Kang, Han Jin, Hao Yang, Jinglin Wang, Shan Gao

**Affiliations:** 1 State Key Laboratory of Pathogen and Biosecurity, Institute of Microbiology and Epidemiology, Academy of Military Medical Sciences, Beijing 100071, China; 15931125585@163.com (X.B.); hcy199701@163.com (C.H.); chen505x@163.com (L.C.); amms_wj@163.com (J.W.); liyanwei_00@163.com (Y.L.); wanwan03012022@163.com (W.W.); jinzhiying96@163.com (Z.J.); lyzaokewenhua@163.com (Y.L.); 7193912@163.com (W.X.); kanglin13011888501@163.com (L.K.); yanghao0065@163.com (H.Y.); wangjlin@bmi.ac.cn (J.W.); 2 School of Basic Medical Sciences, Anhui Medical University, Hefei 230032, China; 3 Institute of Micro-Nano Science and Technology, School of Electronic Information and Electrical Engineering, Shanghai Jiao Tong University, Shanghai 200240, China; jinhan10@sjtu.edu.cn

**Keywords:** ricin, abrin, microfluidic chip, nanoforest structure, biosecurity

## Abstract

Ricin and abrin are phytotoxins that can be easily used as biowarfare and bioterrorism agents. Therefore, developing a rapid detection method for both toxins is of great significance in the field of biosecurity. In this study, a novel nanoforest silicon microstructure was prepared by the micro-electro-mechanical systems (MEMS) technique; particularly, a novel microfluidic sensor chip with a capillary self-driven function and large surface area was designed. Through binding with the double antibodies sandwich immunoassay, the proposed sensor chip is confirmed to be a candidate for sensing the aforementioned toxins. Compared with conventional immunochromatographic test strips, the proposed sensor demonstrates significantly enhanced sensitivity (≤10 pg/mL for both toxins) and high specificity against the interference derived from juice or milk, while maintaining good linearity in the range of 10–6250 pg/mL. Owing to the silicon nanoforest microstructure and improved homogeneity of the color signal, short detection time (within 15 min) is evidenced for the sensor chip, which would be helpful for the rapid tracking of ricin and abrin for the field of biosecurity.

## 1. Introduction

Ricin and abrin are phytotoxins with similar structure and function [1,2] that are classified as Category B bioterrorism agents and included in the Biological Weapons Verification List (BWVL) due to their high toxicity, high hazard, availability, and ease of preparation [3,4]. Ricin is a highly toxic glycoprotein extracted from castor seeds [5,6]; purified ricin has an estimated human lethal dose of 5–10 μg/kg by inhalation or injection [7]. Thus, theoretically, 1 kg of crystallized ricin could kill 3.6 million people. Ricin can be used in assassination or sabotage activities by contaminating food and water sources, through aerosol or spray liquid administration, or by injecting the liquified toxin into the human body with a syringe. For example, an injected ricin pellet is thought to have been used for assassination in the Markov Umbrella Incident [8,9] in 1978, and 20 years ago in the United States, bioterrorist attacks used letters laced with finely powdered ricin, which were sent to prominent government officials [10,11]. According to incomplete statistics, at least 750 cases of ricin-related poisoning and terrorist attacks have occurred since the 1970s, threatening the security and stability of the international community [12]. 

Abrin is a highly effective natural toxin derived from the seeds of acacia [13]. Purified abrin is about 20 to 400 times more toxic than ricin, with an LD_50_ of 0.04 μg/kg in mice and a lethal dose of about 6 μg/kg when ingested by adult humans [14,15]. Abrin poisoning is mostly caused by malicious homicide attempts or accidental ingestion, although a few cases of suicide poisoning have been reported. Due to the hard texture, appealing color, and long-lasting redness of acacia seeds, they are often sold as decorative items, which has led to poisoning caused by accidental ingestion, causing harm to individuals and socioeconomic damage [16]. At present, no specific antidotes exist for either ricin or abrin in clinical practice [17], and the establishment of efficient and sensitive detection methods remains the only essential means by which to reduce the harm caused by toxin poisoning to individuals and society [18].

**Figure 1:**
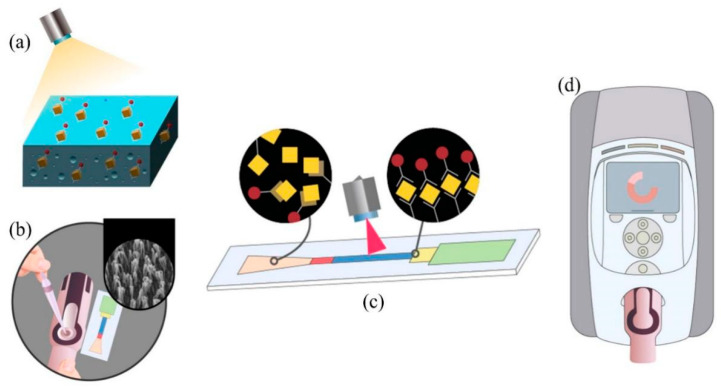
Illustration of the overall experimental strategy. (**a**) The principle of NC membrane action in conventional immunochromatographic test strips. Only the surface signal of the membrane can be measured. (**b**) A microfluidic chip based on nanoforest structure was designed for the detection of AT and RT. (**c**) Progress of the sample reaction. (**d**) The fluorescence signal value can be read after 15 min.

Various detection methods have been developed for ricin and abrin [19], including ELISA [20], liquid chromatography–electrospray ionization tandem mass spectrometry [21], immune-polymerase chain reaction (IPCR) [22], immunochromatographic test strips [23], surface-enhanced Raman spectroscopy (SERS) [24], fiber optic sensors, and sensors based on surface plasmon resonance (SPR) technology [25]. Given the need to make rapid and efficient diagnoses in response to bioterrorist attacks using the toxins or accidental poisoning events, the development of easy-to-operate, on-site rapid detection methods has received widespread attention. The classic of such a test is the immunochromatographic detection method based on the principle of using nitrocellulose membranes (NC) to produce a chromatographic effect for rapid detection of the target [26]. However, only the signal on the surface of the nitrocellulose membrane can be captured when reading signal results, not the reaction signal inside the membrane (Figure 1a). This results in a relatively low detection sensitivity and an inability to perform accurate quantitative analysis of the test substance. In recent years, some studies have improved the performance of chromatographic test strips by changing the type of markers from the earlier use of enzymes, colloidal gold, latex particles, or liposomes to newly developed up-transferred phosphorescent particles, quantum dots, or time-resolved fluorescence markers [27]. Although these new methods have improved the sensitivity to some extent, they still do not solve the problem of signal loss inside the NC membrane (Figure 1a). The microfluidic chip is an emerging detection technology that is gaining importance in field assays, with the advantages of automation, integration, miniaturization, portability, and high throughput [28,29]. However, microfluidic chips often require complex auxiliary pressure systems, leading to expensive overall assay costs and a large instrument size, greatly limiting their application.

Here, we introduce a nanoforest structure to replace NC membranes for chromatography to achieve a highly sensitive target detection rate. The nanoforest structure is an array of fibrous nanostructures with upward protrusions that have an autonomous capillary system capability and the ability to capture target molecules after suitable surface modification [30,31]. The structure has a large surface area that can solidify a large number of capture probes, reducing detection signal loss and allowing quantitative analysis of the target molecules within a limited range. Based on the nanoforest structure, a capillary self-driven microfluidic chip sensor for the detection of ricin and abrin was designed in this study (Figure 1b). The liquid sample flows via capillary driving force in a directed manner, and the molecule to be tested is captured via antibodies in the detection region after binding to the fluorescent probe (Figure 1c). After waiting 15 min to allow time for the reaction, the chip is placed in a portable infrared fluorescence imaging system to read the fluorescence signal value (Figure 1d). This chip solves the problem of signal loss within the NC membrane while avoiding the complex auxiliary system drive challenges required for standard microfluidic chips. As it also improves detection sensitivity and specificity, it shows promise as a new tool for toxin detection with great application prospects. 

## 2. Materials and Methods

### 2.1. Preparation and Characterization of Nanoforest Structures

The nanoforest structure was prepared according to a published method [32].The silicon wafers were ultrasonically cleaned for 15 min in succession with acetone and isopropanol. After being washed with deionized (DI) water and dried under a stream of nitrogen, the silicon wafers were put into a bath of BOE (HF:NH_4_F = 1:6) for another 5 min. After cleaning and drying again, the silicon wafers were dehydrated by baking at 180 °C for 20 min. Then, the surface of silicon wafers was modified by hexamethyldisilazane (HMDS) to combine hydroxy and produced a hydrophobic siloxane structure to enhance the adhesion of photoresist. 

**Figure 2:**
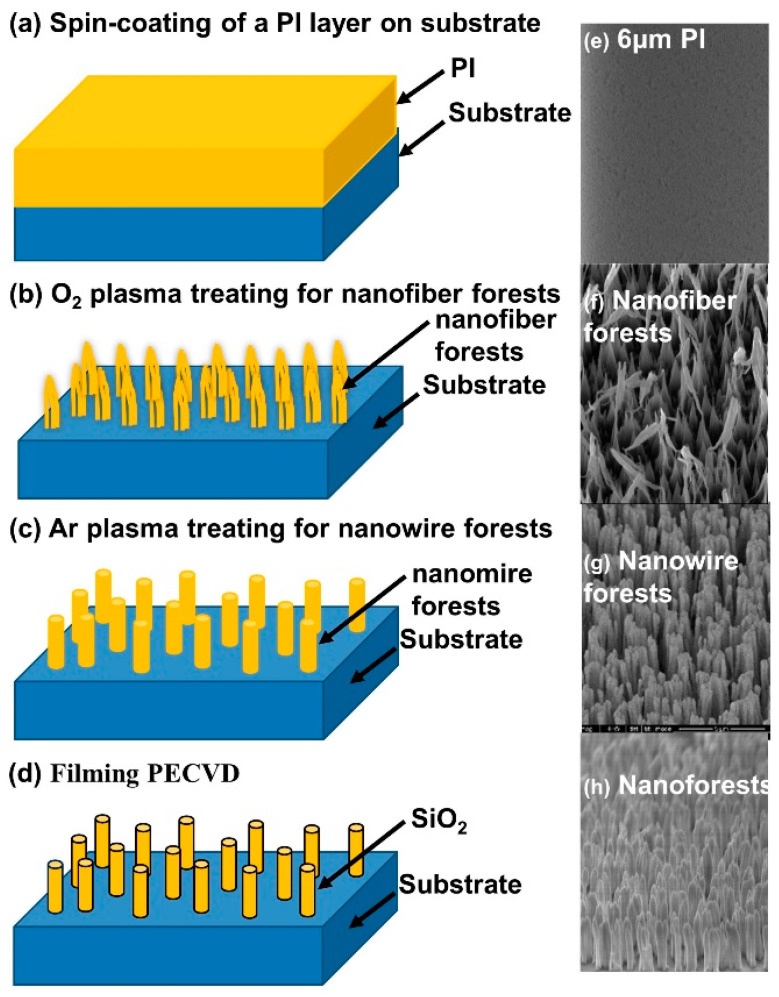
Illustration of the preparation for nanoforest structure: (**a**) process of spin-coating polyimide photoresist; (**b**) O_2_ plasma treatment for nanowire forests; (**c**) Ar plasma treatment for nanowire forests; (**d**) process of SiO_2_ coating by PECVD; (**e**–**h**) SEM images of the PI layer, nanofiber forests, nanowire forests, and nanoforests.

The pre-cleaned silicon wafers were spin-coated with 6 μm polyimide (PI) photoresist. Then, the wafers were placed on the hot plate, baking at 120 °C for 20 min to evaporate organic solvent and solidified surface photoresist (Figure 2a,e). In the experiment of plasma bombardment, the flow rate of O_2_ was 200 sccm (Figure 2b,f), and that of Ar was 150 sccm (Figure 2c,g). The action time was 20 min and 40 min, respectively, and the cavity pressure and power remained at 400 W and 80 m Torr during the whole process. Finally, in order to increase the tolerance, hydrophilicity, and biocompatibility, the Plasmalab System 100 instrument was used to coat SiO_2_ film (Figure 2d,h). The parameters of the coating process were as follows: SiH4 flow 40 sccm, N_2_O flow 800 sccm, NH_3_ flow 40 sccm, temperature range 300 °C, RF power range 200 W.

The nanoforest structure on the prepared chips was observed using a thermal field emission environmental scanning electron microscope (Quanta 400FEG) to measure height, width, density, and specific surface area. Reflectance was tested at wavelengths from 200 to 800 nm using a UV–visible spectrophotometer (Hitachi U-4100). A flow channel with a width of 2 mm and a length of 40 mm was designed and placed at an inclination of 45° to characterize the driving force with the flow rate of PBS solution. 

### 2.2. Surface Modification and Saturation Fluorescence Experiments on Silicon Nanoforest Structured Chips

**Figure 3:**
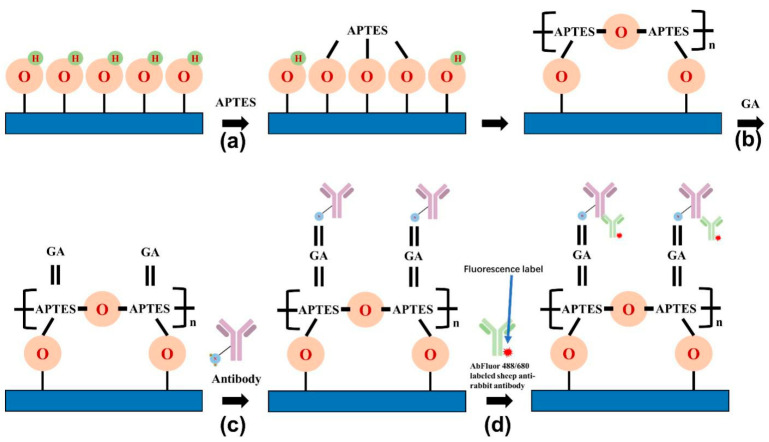
The principle of the saturated fluorescence experiments: (**a**,**b**) Scheme of the surface modification process for the chip. (**c**) The modified nanoforest structure with rabbit antibody added. (**d**) The antibody combined with the sheep anti-rabbit antibody labeled by AbFluor 680 or AbFluor 488.

In order to make the chip acquire the ability to capture proteins, we used the method of aldehyde radicalization, using 3-aminopropyl-trimethoxysiloxyane (APTES) as the silane coupling agent and glutaraldehyde (GA) as the crosslinking agent to modify the surface of the chip [33]. Briefly, the nanoforest structures were cleaned in a 2:1 ratio of H_2_SO_4_/H_2_O_2_ solution for 1 hand then the chips were thoroughly cleaned by sonication in deionized water for 5 min. The cleaned chips were immersed in APTES solution (acetone solution containing 2% APTES by volume) for 20 min and cleaned sequentially with acetone, ethanol, and deionized water and then blown dry under nitrogen (Figure 3a). After that, the aminated chips were then immersed in GA solution (PBS solution containing 5% glutaraldehyde by volume, pH 7.4) for 2 h, cleaned in PBS solution followed by deionized water, and then blown dry under nitrogen (Figure 3b). 

We set saturated fluorescence experiments to compare the structure of nanoforest-structured silicon chips and planar silicon chips. At the beginning, 0.5 μL of rabbit antibodies (1 mg/mL) were added dropwise onto a modified nanoforest structured silicon chip (Figure 3c) and a planar silicon chip, respectively. After that, chips were removed and washed thoroughly with PBS solution containing 1% Tween 20, blown dry under nitrogen, and then placed in PBS solution containing 3% bovine serum albumin (BSA) for 1 h at 37 °C to seal the chips. Then, chips were removed and incubated for 15 min at room temperature in the dark with 0.5 mL of AbFluor 680 and AbFluor 488 labeled sheep anti-rabbit antibody (1 mg/mL, 500-fold dilution) (Figure 3d), washed thoroughly with 1% Tween 20 in PBS solution, and then blown dry under nitrogen. At last, the resulting fluorescence signal was confirmed using the Odyssey infrared fluorescence imaging system and fluorescence microscopy.

### 2.3. Preparation of Microfluidic Chip Sensors

**Figure 4:**
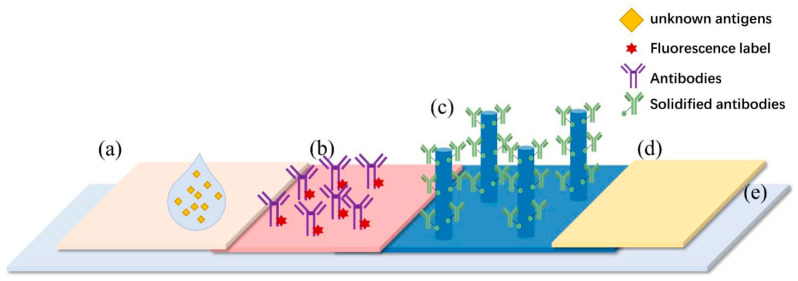
Design of microfluidic sensor based on the nanoforest structure. (**a**) Sample pad. (**b**) Conjugate pad. (**c**) Detection area containing the corresponding capture antibody with nanoforest. (**d**) Absorbent pad. (**e**) The backing plate.

Firstly, to prepare the microfluidic chip sensors for AT and RT detection, the AT and RT polyclonal antibodies (prepared by our laboratory and details can be found in published articles [34]) were sampled at a concentration of 4 mg/mL by the Bio-Chip Injector in the detection area of the surface modified chips, respectively. The amount dotted in each area was 150 nL, and chips were placed in a wet box at 4 °C for incubation overnight. Using the same method as mentioned in Section 2.2, the chip was washed by PBS, Tween 20, and BSA to obtain nanoforest substrates coupled with AT and RT polyclonal antibodies (Figure 4b). Then, the prepared chips were used in the assembly of sensors.

The sample pad was a glass cellulose film with a 4 mm upper bottom and a 15 mm lower bottom in a trapezoidal shape (Figure 4a). The conjugation pad was also a glass fiber membrane with a size of 4 mm × 2 mm and a solidified fluorescently labeled probe (Figure 4b). The reaction film was a nanoforest substrate chip, which was coupled with polyclonal antibodies to toxins (Figure 4c). In addition, the absorbent pad was a paper made by absorbent materials (Figure 4d). In addition, the resin material was produced by 3D printing to reduce the cost. Those components were successively pasted on the backing plate (Figure 4e), with each component overlapping 1 mm. The assembled microfluidic chip sensors were sealed and stored at 4 °C.

### 2.4. Microfluidic Chip Sensors Signal Measurement

We evaluated sensitivity of the sensors by obtaining the calibration curves established by the fluorescence intensity of AT and RT at different concentrations and in different simulated samples. A series of gradient dilutions of both toxin antigens were first created using 0.01 M PBS solution; 3% BSA was used as a blank control for the toxin. Aliquots of 100 μL each of the blank control and the toxin diluted to 2 pg/mL, 10 pg/mL, 50 pg/mL, 250 pg/mL, 1.25 ng/mL, and 6.25 ng/mL were added dropwise to the sample pads of sensors (Figure 1b). After 15 min, the signals were read using the Odyssey infrared fluorescence imaging system (Figure 1d). The detection limits were quantified using fluorescence intensity as the evaluation index and two standard deviations ($\bar{x}\pm 2s$) as the threshold value. The toxin was then diluted to the above gradient using juice and milk as mock samples. According to the fluorescence intensity, the calibration curves were established to determine the detection limit of the chip and to verify the ability of the sensor to detect complex matrices. In experiments to evaluate the specificity of the sensors, 10 ng of *Clostridium perfringens* ε toxin (ETX [35]) and botulinum toxin (BoNT/A [36]) (prepared by our laboratory and details can be found in published articles) were used as unknown samples added to the sample pads of sensors. The fluorescence intensity values were read at the end of the reaction and the calibration curves were also established. 

## 3. Results and Discussions

### 3.1. Characterization of Nanoforest Structure Chip

**Figure 5:**
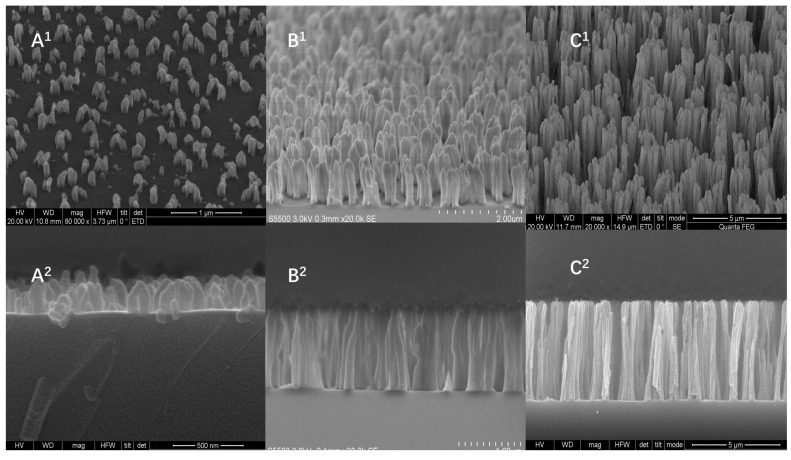
SEM photographs of nanoforest structures (1,2 are the same nanoforest at a different angle). (**A^1^**) 2 μm, 45°. (**A^2^**) 2 μm, 90°. (**B^1^**) 5 μm, 45°. (**B^2^**) 5 μm, 90°. (**C^1^**) 10 μm, 45°. (**C^2^**) 10 μm, 90°.

The core of this sensor design lies in the special nanoforest structure chip in the reaction membrane part, so the characteristics of this structure are discussed to make it work better. To obtain a larger surface area and allow nanoforest structures to bind more fully to the trapped proteins, we explored results from preparation parameters using scanning electron microscopy. Scanning electron microscopy at different photoresist thicknesses revealed that the period, diameter, and morphology of the nanofiber structure could be tuned by controlling the thickness of the photoresist coating, the type of plasma source, the plasma bombardment time, the gas flow rate, the pressure, and the power. Scanning electron microscopy (SEM) results (Figure 5) and measurement parameters (Table 1) show differences in nanoforest structure morphology depending on photoresist coating thickness. With further increases in the photoresist thickness beyond 10 µm, the nanofibers appeared to be inverted and did not form a complete structure. Nanoforest structures with different morphologies have different abilities to capture protein molecules; the larger the depth-to-width ratio the better the surface area can fully capture antibodies, increasing the antibody loading. To maximize antibody loading, the nanoforest chip with 10 μm photoresist thickness was selected for use in subsequent experiments.

**Figure 6:**
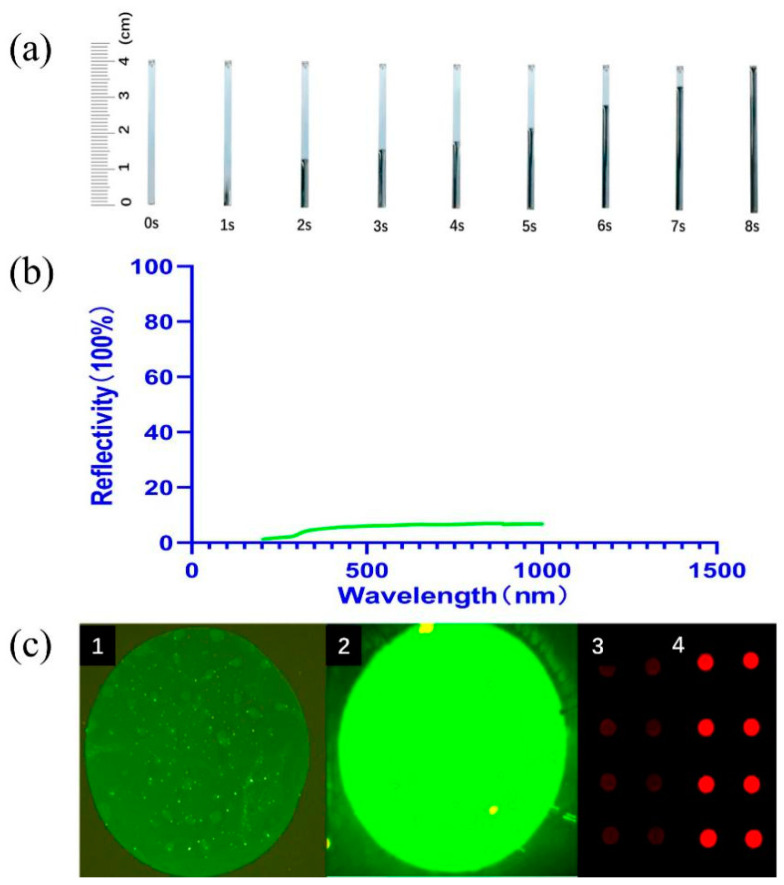
Characterization of nanoforest structure. (**a**) Flow rate test of silicon nanoforest chip recorded in 1 s intervals. The flow speed of PBS was recorded by the distance traveled per second. (**b**) Reflectance for nanoforest structure on silicon substrate. (**c**) Scheme of the silicon nanoforest chip under fluorescence microscope: (1) silicon-based plane in 488 nm wavelength; (2) nanoforest structure in 488 nm wavelength; (3) silicon-based plane in 688 nm wavelength; (4) nanoforest structure in 688 nm wavelength.

To verify that the chip had good detection potential, we characterized the chip by its self-driving capability, optical properties, and protein adsorption efficiency. The absence of an additional driver makes our assay device smaller and more portable, facilitating in situ detection. Flow rate tests were performed on the silicon substrate chip flow channel to verify if the capillary self-driving force of the nanoforest structure was appropriate. The flow rate of PBS reached 5 mm/s (40 mm, 8 s) in the flow channel prepared with the nanoforest structure and without external force (Figure 6a). Thus, the flow rate of the nanoforest chip is faster and requires lower sample volume than the conventional nitrocellulose membrane. The tiny pore size and good hydrophilicity of the structure allow the chip to create a driving effect without additional modifications, reducing the difficulty of surface treatment and simplifying antibody labeling. In addition, to enable the optical reader to capture more signals, we performed reflectance tests on the prepared silicon nanoforest structure to verify its optical properties. The overall value of reflectance was low for detection in the wavelength range from 200 to 1000 nm but increased with increasing wavelength (Figure 6b). The lower reflectance indicates better light absorption and less loss of incident light, i.e., the excitation light can be better absorbed by the fluorescent dye and converted into emitted light. The nanoforest structure had a low reflectance, indicating that it can be used to prepare chips with good optical properties, which improves sensitivity of detection. At the same time, since the silicon substrate itself is impervious to light, designing the optical path of the detection instrument has fewer design requirements, thereby lowering instrument development costs.

Finally, to immobilize more proteins on the chip surface and improve detection efficiency, we performed saturated fluorescence experiments on planar silicon wafers and nanoforest microfluidic chips to compare protein immobilization efficiency (Figure 6c). After labeling with AbFluor 488 or AbFluor 680 fluorescent antibodies, the surface modification of the silicon-based planar structures lasted for a short time with low saturated fluorescence color rendering intensity, while the fluorescence signal on the surface of the nanoforest structures was significantly enhanced. This is because the nanoforest structure provides more binding sites per unit space due to its relatively large surface area compared to the planar structure, allowing the surface to bind more antibody molecules. In addition, we experimentally verified that the structure showed good color development using fluorescent dyes of two different wavelengths. However, due to instrument limitations, the detection of green fluorescence using fluorescence microscopy could provide only qualitative results, not quantitative ones. Therefore, for statistical analysis, fluorescence intensity values in our subsequent tests used AbFluor 680 red dye, and the results were read by an infrared fluorescence imaging system.

After characterizing the nanoforest structure chip, our results indicate that the antigen and antibody can react both on its surface interior, and the fluorescence signal can be read completely. In addition, the nanoforest structure has good protein adsorption and capillary self-driving force, which can immobilize specific target molecules while directing the flow of samples and assay binders to the reaction area. The improved protein adsorption capability allows the nanoforest structure to immobilize more target molecules, thus reducing signal loss and improving sensitivity, while the strong self-driving property makes it possible to complete the reaction without requiring additional driving devices. 

### 3.2. Sensor Detection of Two Plant Toxins 

**Figure 7:**
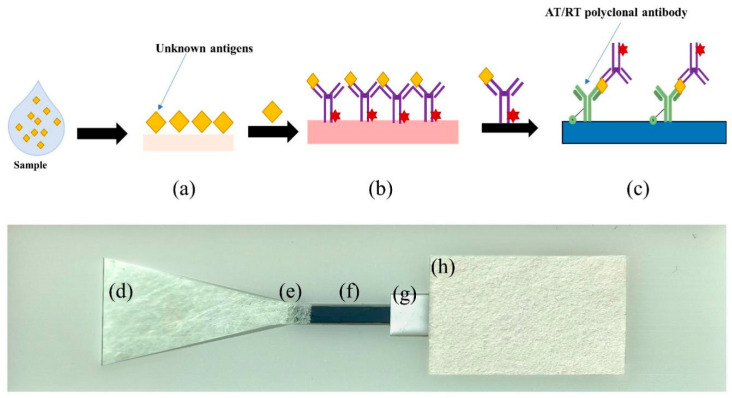
Physical assembly and flow process of the microfluidic chip sensors. (**a**,**d**) Sample pad. (**b**,**e**) Conjugate pad. (**c**,**f**) Detection area containing the corresponding capture antibody with nanoforest. (**g**) Speed-limiting membrane. (**h**) Absorbent pad.

After completing characterization of the nanoforest-based structure, we used the nanoforest structure as the detection region for the microfluidic chip sensors. After adding a drop of liquid containing the toxin protein onto the sample pad (Figure 7a,d), the large particulate matter was filtered out through the pad and the remaining liquid sample re-solvated within the conjugate pad, free of the fluorescently labeled probe, which remained solidified within the pad (Figure 7b,e). The fluorescently labeled probe bound to the target molecule in the liquid sample, automatically flowed directionally, and solidified in the nanoforest detection area containing the corresponding capture antibody (Figure 7c,f), while the waste liquid continued to flow into the absorbent material of the sample pad (Figure 7h). After surface functionalization with amination and glutaraldehyde, the detection region can be solidified with antibodies that specifically capture the target molecule to be detected to quantify results. In practice, the detection results are compromised by poor antigen–antibody binding because the flow rate is too fast. Therefore, during assembly of the microfluidic chip, we placed a small amount of NC film between the nanoforest substrate and the absorbent film as a rate-limiting barrier material (Figure 7g) to reduce the flow rate of the liquid and prolong the antigen–antibody reaction time. In addition, the use of conventional materials such as NC membranes, sample pads, and 3D-printed resin substrates effectively reduces the manufacturing cost and facilitates the further industrial development of microfluidic chips. 

**Figure 8:**
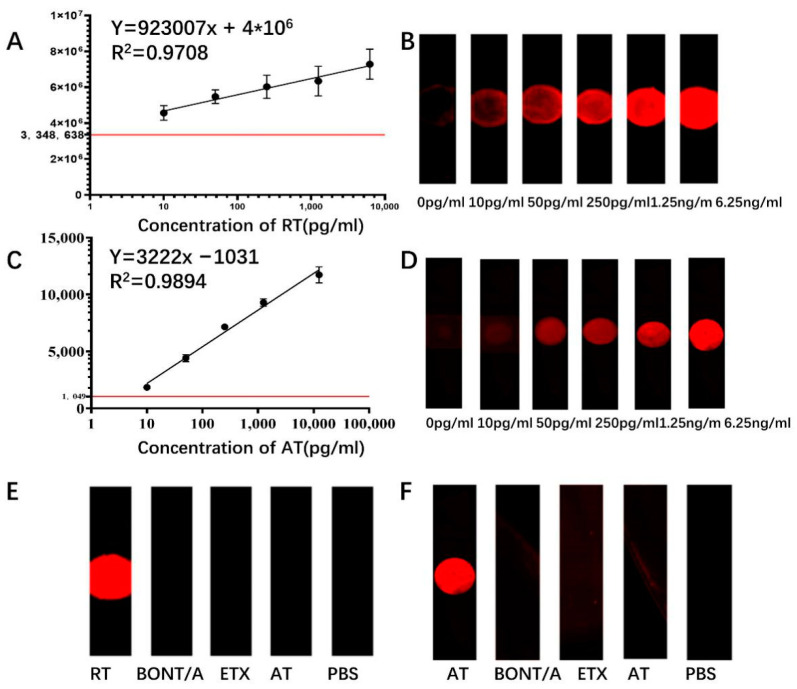
(**A**) Dose–response curve for the RT sample at different concentrations. The x-axis denotes the toxin concentration (pg/mL), and the y-axis denotes the fluorescence intensity at 700 nm. The red horizontal line represents the boundary between negative and positive. (**B**) Fluorescence response image at corresponding concentrations for RT. (**C**) Dose–response curve for the AT sample at different concentrations. (**D**) Fluorescence response image at corresponding concentrations for AT. (**E**) Detection of cross-reaction between RT and three other toxins. (**F**) Detection of cross-reaction between AT and three other toxins.

We completed the preparation of sensors for ricin and abrin detection based on the above microfluidic chip and verified the detection capability of the sensors by evaluating their sensitivity and specificity. First, the calibration curves were established by setting a concentration gradient for ricin. The fluorescence signal intensity increased with the increase of the concentration of the toxin added dropwise. When the toxin concentration ranged from 10–6250 pg/mL, the trend line of the ricin standard was y = 923,007x + 4 × 10^6^, where y denotes the fluorescence intensity at 700 nm and x denotes the toxin concentration (pg/mL). The results for abrin were similar to those for ricin with a trend line of y = 3222x − 1031 (Figure 8A). Thus, our new chip improves detection sensitivity and significantly reduces the time required for detection. Given the high homology of AT and RT, we next validated the specificity of this method by introducing botulinum toxin (BoNT/A) and Clostridium perfringens ε toxin (ETX) as controls against blank samples for evaluation. Only the corresponding toxin was recognized by the sensor to produce a specific fluorescence signal; the fluorescence intensity of the non-target toxins did not differ from the fluorescence values of the blank samples (Figure 8E,F). Thus, the nanoforest structure-based microfluidic chip sensor has good specificity for the detection of RT and AT without cross-reactivity. Compared with the ELISA method and the colloidal gold immunochromatographic test strip method established in our laboratory, this method improved the detection limit of both toxins by approximately 1000-fold, allowing detection of amounts as small as 10 pg/mL. In contrast, the sensitivity of the current colloidal gold immunochromatographic test strip for ricin was only 0.5 ng/mL [37]. In addition, compared with the other current methods reported in the literature, the time-consuming and instrument cost are optimal under the same level of sensitivity, as shown in Table 2.

**Figure 9:**
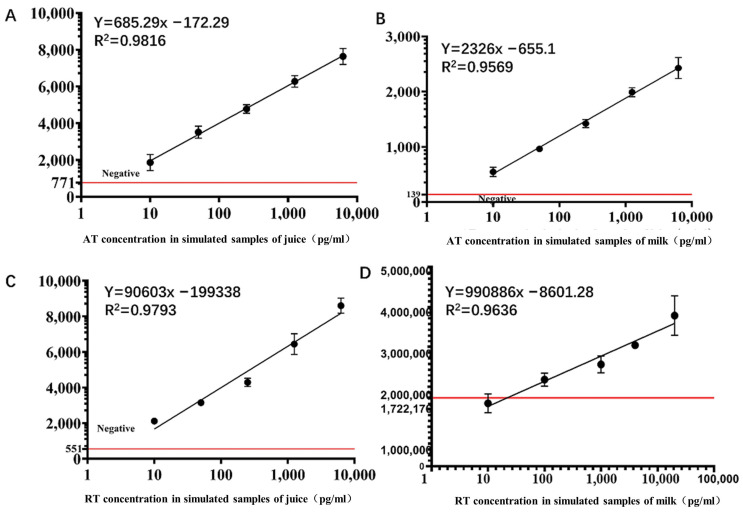
Fluorescence detection curves of ricin and abrin in different food samples. (**A**) AT samples in juice. (**B**) AT samples in milk. (**C**) RT samples in juice. (**D**) RT samples in milk. The red horizontal line represents the boundary between negative and positive results.

To further validate the ability of the sensor to detect the toxins in realistic applied cases, we set up two mixed toxin samples, with juice and milk, to validate the detection capability of this chip based on the premise that food poisoning is closer to a potential bioterrorist attack scenario and that accidental poisoning occurs in practice. In the juice sample, sensitivity was not affected and remained below 10 pg/mL, with good linearity in the range of 10–6250 pg/mL (Figure 9A,C). However, for the RT sample in milk, the sensitivity of the chip decreased from 10 pg/mL to 40 pg/mL, while still showing good linearity in the range of 50 pg/mL to 6250 pg/mL (Figure 9B,D). This may reflect an effect of milk on the sensitivity of the chip; nonetheless, the results indicate that the chip remains valid for complex matrices.

## 4. Conclusions

In this study, a microfluidic chip sensor with a capillary self-driven capability based on nanoforest structure was successfully prepared and found to be effective for the rapid detection of two phytotoxins in the field. The concentration of either phytotoxin in a sample can be obtained sensitively and accurately within 15 min using this method, with a lower detection limit of 10 pg/mL and a linear range from 10–6250 pg/mL. Importantly, the simple structure, easy operation, and high sensitivity of the sensor overcomes the difficulties of poor color signal homogeneity and difficult quantitative detection that occur with current immunochromatographic test strips or the complex structure and high cost of other microfluidic chips. This new microfluidic chip sensor thus provides a good foundation for future related research and product development.

## Figures and Tables

**Table 1:** The density, diameter, and height of nanoforest structures for different photoresist thicknesses.

Photoresist Thickness (μm)	Density (Fibers/μm^2^)	Diameter (nm)	Height (μm)
2	10	100	0.2
5	15	50–100	1.8
10	20	200–400	5

**Table 2:** Comparative analysis of several different detection methods for AT and RT.

Name	Detectable Toxin Agents	Sensitivity	Time	Cost of Equipment
ELISA	RT [38]	0.093 ng/mL	4–5 h	Expensive
	AT [39]	1 ng/mL		
Immunochromatographic Test Strip	RT [40]	0.5 ng/mL	15 min	Cheap
	AT [41]	3 ng/mL		
MALDI-TOF MS	RT [42]	0.2 ng/mL	3.5–7.5 h	Expensive
	AT [43]	40 ng/mL		
Electrochemical Luminescence Method	RT [44]	0.2 ng/mL	5.5 h	Expensive
	AT [45]	5 pg/mL		
This work	RT, AT	10 pg/mL	15 min	Cheap

## Data Availability

Not applicable.
